# Review Article of Platelet-Rich Plasma and Facet-Mediated Mechanical Low Back Pain

**DOI:** 10.3390/biomedicines14102174

**Published:** 2026-09-25

**Authors:** Aaron Chang, Mitchell Doerr, Justin Dzierzawski, Oluseun Olufade, Di Cui

**Affiliations:** 1 Department of Physical Medicine and Rehabilitation, Emory University School of Medicine, 1441 Clifton Road NE, Atlanta, GA 30322, USA; oolufad@emory.edu (O.O.); di.cui@emoryhealthcare.org (D.C.); 2 Department of Family Medicine, Corewell Health West, 100 Michigan St. NE, Grand Rapids, MI 49503, USA; mitchell.doerr@corewellhealth.org; 3 Department of Physical Medicine and Rehabilitation, Emory University School of Medicine, 80 Jesse Hill Jr Dr SE, Atlanta, GA 30303, USA; jtdzier21@gmail.com; 4 Department of Orthopedics, Emory University School of Medicine, 80 Jesse Hill Jr Dr SE, Atlanta, GA 30303, USA; 5 Department of Orthopedics, Emory University School of Medicine, 1441 Clifton Road NE, Atlanta, GA 30322, USA

**Keywords:** orthobiologics, lumbar spine degeneration, mechanical low back pain, regenerative medicine, spine injections, platelet-rich plasma

## Abstract

Introduction and Aim: Facet-mediated mechanical low back pain is a common contributor to chronic disability worldwide. While platelet-rich plasma (PRP) has demonstrated efficacy in discogenic low back pain, its role in facet joint-mediated pain remains less clearly defined. This narrative review aimed to evaluate the existing clinical evidence on the use of PRP for lumbar facet-mediated mechanical low back pain. Materials and Methods: A comprehensive PubMed search was conducted in October 2024 for studies published between 2014 and 2024, with one additional eligible study published in 2025 included subsequently. Eligibility included clinical studies evaluating intra-articular or periarticular PRP directed at lumbar facet-related pain. Because the original search was limited to a single database and the review was not conducted under a prospectively registered systematic-review protocol, the present article is explicitly framed as a structured narrative review rather than a systematic review. Results: Six studies were identified: one randomized controlled trial, one prospective controlled nonrandomized study, two prospective case series, one retrospective observational study, and one small case series. The most direct comparative evidence suggests that PRP may provide longer-lasting benefit than local anesthetic/corticosteroid injection, while the 2025 prospective nonrandomized study suggests longer-term improvement compared with local anesthetic. However, several studies used multiple spinal injection targets, and the evidence is further limited by small samples, heterogeneous PRP formulations and treatment protocols, variable diagnostic criteria, and incomplete safety reporting. Conclusion: Overall, current evidence supports PRP as a promising but not established treatment for lumbar facet-mediated low back pain. Nevertheless, the overall quality of evidence remains limited, underscoring the need for larger, standardized, and long-term controlled studies.

## 1. Introduction

Low back pain is an increasing global issue. According to the Global Burden of Disease (GBD) study, the number of years lived with disability (YLDs) due to low back pain increased from 58.2 million in 1990 to 83 million in 2010 [1]. More recent estimates from 2020 indicate that low back pain affected approximately 619 million people globally [2]. In the United States alone, low back pain is among the most expensive health condition, with an annual expenditure of $134.5 billion in 2016 [3].

Mechanical low back pain arises intrinsically from the spine’s structural and functional components. The etiology encompasses a spectrum of disorders including lumbosacral muscle strain, intervertebral disc degeneration or herniation, lumbar spondylosis, spondylolisthesis, facet joint osteoarthritis, spinal stenosis, and traumatic injury. Importantly, these conditions often coexist and interact biomechanically; alterations in one anatomical component affect the overall load distribution and degenerative progression throughout the lumbar three-joint complex. Clinically, this pain is reproducible, varies predictably with movement and posture, and typically improves with activity modification or specific directional movements, whereas non-mechanical low back pain may arise from systemic etiologies such as infection, malignancy, inflammatory disease, nephrolithiasis, or referred visceral pathology, and is distinguished by constitutional symptoms, progressive neurological deficits, and pain patterns independent of mechanical factors. Facet-mediated low back pain comprises a significant percentage of patients experiencing low back pain.

Typically, facet mediated low back pain is initially managed with conservative measures, including medication, rehabilitation, and interventional procedures. Interventional procedures such as corticosteroid injection, medial branch blocks and radio-frequency ablations have demonstrated varying but overall favorable outcomes. When conservative measures are less than effective, surgical treatment can be pursued [4]. Surgical spinal fusion is often indicated in cases of high instability or degeneration of the spine. However, the incidence of pseudoarthrosis in long instrument posterior spinal fusions is estimated to be from 25–35% [5]. In addition, adjacent level degeneration is also an undesirable sequela of spinal fusion.

Regenerative medicine also has recently emerged as a treatment option for low back pain, with platelet rich plasma (PRP) and other orthobiologics demonstrating repair to damaged tissues [4]. PRP is created by concentrating platelets from a patient’s whole blood through centrifugation, allowing for concentration of platelets 5–10 times above baseline levels. These supraphysiologic concentrations contain many growth factors such as Platelet-Derived Growth Factor (PDGF), Transforming Growth factor-β (TGF-β), Insulin-Like Growth Factor (IGF), Vascular Endothelial Growth Factor (VEGF) and Epidermal Growth Factor (EGF), all of which have been shown to play important roles in healing [6].

There has been a great amount of research done on PRP and intradiscal low back pain therapy that has shown promising results. However, there has been limited research on PRP and facet-mediated pain. This narrative review set out to evaluate the effects of platelet rich plasma injection on mechanical low back pain.

## 2. Materials and Methods

### Literature Search and Selection

A comprehensive literature search was conducted using PubMed in October 2024. A professional librarian experienced in health science literature search assisted with guiding and formulating the search strategy. The original PubMed strategy consisted of three conceptual components: mechanical/non-discogenic low back pain, biologic/PRP injection terminology, and injection-target terminology. The search was: (“low back pain” OR lumbar) AND (“non-discogenic” OR “mechanical pain”) AND (“biologic injections” OR PRP OR “platelet rich plasma” OR “platelet poor plasma” OR “stem cells” OR “intramuscular injections” OR “intraarticular injections” OR “cell component” OR periarticular) AND (intramuscular OR intraarticular). Reference Table 1 for complete search strategy. Studies were considered eligible when they: (1) enrolled human participants with chronic low back pain attributed, at least in part, to lumbar facet pathology or facet-joint syndrome; (2) evaluated autologous PRP; (3) delivered PRP by intra-articular or periarticular injection to lumbar facet-related structures, either as the primary target or as one component of a clearly described multi-target intervention; and (4) reported clinical outcomes such as pain, disability, function, satisfaction, or quality of life. Randomized trials, controlled observational studies, cohort studies, and case series were considered.

Only articles from 2014 to 2024 were included in the search. Using the created search string in PubMed, the search term yielded 103 articles. After reviewing the titles and abstract of the articles it revealed 15 potential articles. From there, the 15 articles were assessed for full-text eligibility. After reviewing the remaining articles, 5 were identified that were considered relevant to the review. The original literature search was conducted in PubMed in October 2024 and was limited to studies published between 2014 and 2024. Following completion of the original search, the Principal Investigator identified a newly published study by Baltzer et al. (2025) [7] that was directly relevant to the review question. The study was subsequently reviewed by the authors and determined to meet the predefined eligibility criteria; therefore, it was included in the final review. This study was not identified through an updated database search and was added separately to ensure that the most recent directly relevant clinical evidence was considered. The authors acknowledge that this approach does not constitute a formal updated literature search and represents a limitation of the review. Its identification through investigator awareness rather than a reproducible search process may introduce selection bias. Consequently, the final set of included studies should be considered a structured, clinically relevant evidence sample rather than a comprehensive representation of all available evidence.

Studies were not treated as direct evidence for facet-joint efficacy when PRP was delivered only to unrelated spinal structures. Importantly, studies in which PRP was injected into multiple structures (for example, the facet-joint, disc, epidural space, and/or paravertebral musculature) were retained because they were relevant to the broader clinical question but were classified as indirect or confounded evidence for facet-specific efficacy.

## 3. Results

The PubMed search identified literature addressing the use of PRP for lumbar facet-mediated low back pain. Six clinical studies were considered relevant to the focus of this narrative review, including case series, observational studies, a randomized controlled trial, and a prospective controlled nonrandomized study. Reference Table 2 for a summary of the studies.

Aufiero et al. in 2015 [6] published a case series of five patients who each received a series of three PRP injections into facet joints and surrounding ligaments. Patients were selected based on history, physical examination, and lack of significant disc pathology on MRI. Each injection was spaced out 4–6 weeks apart. Outcomes were measured using the Visual Analog Scale (VAS), a subjective rating of one’s pain on a scale of 0–10, with a higher score signifying a more severe pain level, and a subjective survey that included symptom relief and functional improvements. Follow-up examinations range from 6 to 12 months. Of note, not all patients were treated for low back pain, case 1 and case 5 were treated for cervical pain. Cases 2, 3 and 4 were treated for lumbar pain. Case 2 showed at least 30% improvement following the first injection and 60% improvement following the second series. At the 9-month follow-up, patient-reported pain level was 1/10 after completing three PRP injections, compared with 6/10 at baseline. Case 3 showed 40% pain relief following the second injection. At the 12-month follow-up, he ranked his pain 2/10, which was previously rated 4/10–6/10 for pain. Lastly, Case 4 showed a 70% improvement following their third injection.

Kirchner et al. in 2016 [8] was an retrospective observational study. In this study, 86 patients with history of chronic low back pain and degenerative disease of the lumbar spine received one intradiscal, one intra-articular facet, and one transforaminal epidural injection of plasma rich in growth factors (PRGF-Endoret). Pain was assessed using the VAS after the procedure at one-, three-, and six-months post-procedure. Results demonstrated a statistically significant decrease from pre-procedure treatment 8.4 ± 1 to 4 ± 2.6 at one month, 1.7 ± 2.3 at two months, and 0.8 ± 1.7 at six months post-procedure, suggesting that PRGF offers improving relief as time from the procedure increased.

Wu et al. in 2016 [9] was a case series which assessed the efficacy of platelet rich plasma in patients who had low back pain secondary to degenerative changes in facet joints. In the study, 19 participants with lumbar facet joint syndrome were enrolled. Patients received a lumbar facet joint injection with autologous PRP under X-ray fluoroscopic control. Patients followed up at one week, one month, two months, and three months following treatment. The primary outcome was measured by low back pain VAS at rest and during flexion, Roland Morris disability questionnaire, Oswestry disability index (ODI), and MacNab criteria. VAS scores showed a continued decrease at three months. The Roland–Morris Disability Questionnaire (RMDQ) measures functional disability as it pertains to low back pain, with a higher score indicating greater disability. The Oswestry Disability Index (ODI) evaluates the degree of disability caused by low back pain across multiple domains, with higher values reflecting more severe disability. Mean RDQ and ODI showed significant reduction in post treatment of patients at three months. Lastly, the Modified MacNab Criteria provide an assessment of surgical outcome, with the patient assigned to one of four categories (excellent, good, fair, or poor) based on pain relief and return to normal activity. Clinically, the outcomes were favorable, with 79% of patients reporting good or excellent improvement at the three month follow up.

In 2017, Wu et al. [10], published a prospective randomized trial. A total of 46 participants with lumbar facet joint syndrome were divided into two groups. Group A received intra-articular PRP injection and Group B received intra-articular local anesthetic/corticosteroid injection. Patients were evaluated by the VAS at rest and during flexion, as well as the RMDQ, ODI, and modified MacNab criteria. Patients were evaluated immediately post injection, one week, one month, two months, three months, and six months post intervention. Both groups demonstrated initial statistical improvements in VAS, RMDQ, and ODI scores. However, the durability of relief differed significantly between cohorts. Group B peaked at one month with high satisfaction (85%) but declined rapidly to 20% objective success by six months. In contrast, Group A demonstrated a trajectory of gradual, sustained improvement, with satisfaction and success rates continuing to rise over the six-month period, suggesting PRP offers superior long-term efficacy compared to steroids.

Machado et al. in 2022 [11] published a prospective case series using a multi-target PRP injection approach for low back pain. A total of 46 patients were injected with PRP into the facet joints, intervertebral discs, epidural space and/or paravertebral muscles, depending on the patient presentation, physical examination and imaging studies. The VAS and RMDQ were used for evaluation, with evaluations at pretreatment (VAS only), 12-, 26- and 52-weeks. The results showed that the VAS mean from beginning to 12 weeks was reduced from 8.48 to 5.17 and the mean RMDQ from 18.0 to 10.98 at 12 weeks (*p* < 0.001). These statistically significant improvements in ratings were sustained over 52 weeks.

Baltzer et al.’s 2025 [7] prospective controlled study evaluated the efficacy of CT-guided periarticular injections (PAT) for lumbar facet joint pain in 78 patients who self-selected into either the autologous leukocyte-poor Platelet-Rich Plasma (PRP) group (*n* = 59) or the local anesthetic (LA/bupivacaine) group (*n* = 19), both receiving a series of 3–5 weekly injections for an average of 3.3 and 3.53 repetitions respectively. The primary outcome measures included VAS, the ODI, and the Veteran RAND-12 (VR-12), a health-related quality of life survey that produces a Physical Composite Score (PCS) and a Mental Composite Score (MCS), with scores standardized to a mean of 50 in the U.S. general population, with scores below 50 indicating a higher health burden. While both groups saw comparable pain score reductions two weeks after injection (PRP 5.93 to 4.36; LA: 6.05 to 4.53), the PRP group demonstrated a sustained, statistically significant benefit over the LA group across all primary outcomes at 12 months. Specifically, the PRP group’s average pain score dropped to 2.81 (*p* < 0.001), while the LA group returned to 4.25 (not statistically significant). Similarly, the PRP group’s ODI score dropped significantly from moderate disability (23.46) to minor disability (13.65, *p* < 0.001), while the LA group’s drop to 14.38 did not reach statistical significance. Finally, the VR-12 at 12 months showed the PRP group improved both its PCS (38.6 to 43.0) and MCS (48.4 to 52.7), whereas the LA group improved its PCS (34.8 to 42.0) but failed to sustain its mental health quality of life, with the MCS slightly decreasing (45.5 to 45.0).

## 4. Discussion

The principal finding of this review is not that platelet-rich plasma (PRP) has been established as an effective treatment for lumbar facet-mediated low back pain, but rather that the limited clinical literature suggests a potentially durable benefit in selected patients. This distinction is important because only one included study was randomized, the second comparative study was nonrandomized and patient-selected, and several studies lacked a comparator. In addition, two studies used multi-target injections, limiting the ability to attribute observed improvements specifically to the facet joint. Taken together, the current evidence is encouraging but remains insufficient to establish PRP as a standard treatment for facet-mediated low back pain.

The most informative evidence regarding comparative efficacy comes from the 2017 randomized trial by Wu et al. [10]. Both PRP and local anesthetic/corticosteroid injections were associated with early improvement in pain, but improvement was maintained for a longer period in the PRP group. This finding raises the possibility that PRP may offer an advantage in durability rather than immediate analgesia. Such a pattern is biologically plausible because local anesthetic and corticosteroid injections can provide relatively rapid symptom relief, whereas PRP is hypothesized to act through slower modulation of local tissue and inflammatory signaling. However, the study does not establish superiority of PRP across all outcomes or time points. The 2025 prospective controlled study by Baltzer et al. [7] provides additional support for a potential durability benefit, but its nonrandomized design requires caution. The results showed that, at 12 months, VAS scores and ODI scores were sustained compared to local anesthetic. However, participants selected their treatment, producing a marked imbalance between treatment groups, and patient preference may have been associated with expectations, prior treatment experiences, socioeconomic factors, or other characteristics that also influence outcomes. Therefore, the findings from Baltzer et al. [7] are best interpreted as supportive prospective observational evidence rather than as equivalent to randomized comparative evidence.

The potential durability of PRP is also consistent with its proposed biological mechanism. PRP contains platelet-associated mediators and growth factors that may influence extracellular matrix signaling, cellular migration, tissue remodeling, angiogenic pathways, and inflammatory responses [12]. These effects provide a plausible rationale for a treatment whose benefits may develop more gradually than those of local anesthetic-based interventions. However, the biological activity of PRP is unlikely to be determined by platelet concentration alone. Leukocyte content, platelet activation, red-cell contamination, injectate volume, preparation method, and the target tissue may all influence the biologic profile of the final product [4,13]. Thus, the apparent clinical benefit of PRP should be considered in the context of how the preparation was produced and administered rather than as a uniform treatment effect across all PRP products.

The heterogeneity of PRP preparations in the included studies highlights this issue. Baltzer et al. [7] used leukocyte-poor PRP, while Kirchner et al. [8] used plasma rich in growth factors with leukocyte-poor characteristics, and Machado et al. [11] also described a leukocyte-poor preparation. In contrast, the Wu studies [9,10] provide limited characterization of leukocyte content. Because no included study directly compared leukocyte-rich and leukocyte-poor PRP for lumbar facet-mediated pain, the available evidence cannot establish whether one preparation is superior. In a 2024 in vitro and in vivo study of intervertebral disc degeneration, Zhang et al. [14] found that leukocyte-poor PRP (Lp-PRP) produced greater extracellular matrix synthesis and reduced apoptosis compared with leukocyte-rich PRP (Lr-PRP), whereas Lr-PRP was associated with increased catabolic activity. Ultimately, the study concluded that Lp-PRP had better effects than Lr-PRP in repairing intervertebral disc degeneration. These findings suggest that leukocyte content may be an important determinant of PRP activity, although the study evaluated intervertebral disc degeneration rather than facet-mediated pain and therefore cannot be directly extrapolated to facet joint injections. Future studies should compare Lr-PRP to Lp-PRP to help establish which is superior in facet arthropathy. Furthermore, lack of standardization in preparing PRP represents the most critical barrier to clinical translation, as preparation protocols vary substantially across studies and commercial kits, with centrifugation speeds ranging from 1500–3000 rpm and platelet concentrations varying from 500,000 to 1,500,000/μL [4]. Table 3 shows how much the protocol as well as concentration of PRP can vary. The variation in PRP composition directly impacts therapeutic efficacy and limits the ability to compare outcomes across studies. Future studies should address the different concentrations of PRP as this can have a large impact on the efficacy of PRP. When treating knee osteoarthritis with PRP, it has been shown that higher concentrations of PRP have been associated with better clinical outcomes. Berrigan et al. [15], 2025 examined 29 studies with a total of 31 arms for using PRP to treat knee osteoarthritis. It was found that 28 of the arms had a positive outcome and the mean concentration of PRP was 5500 ± 474 × 10^6^. The three arms that had no positive outcomes had a mean platelet dose of 2302 ± 437 × 10^6^.

An additional limitation of the available literature is the incomplete reporting of diagnostic criteria used to identify facet-mediated pain. Aufiero et al. [6] selected patients based on clinical history and physical examination, with MRI used to exclude significant disc pathology. Wu et al. [9,10] specifically enrolled patients described as having lumbar facet joint syndrome and performed fluoroscopically guided intra-articular facet injections. However, the included studies did not consistently report standardized diagnostic blocks or uniform criteria incorporating clinical assessment, imaging findings, and/or controlled diagnostic injections. This variability introduces potential diagnostic heterogeneity and makes it difficult to determine whether all treated patients had facet joints as the primary pain generator. Consequently, differences in diagnostic methodology should be considered when interpreting the reported treatment effects and may limit the generalizability of the findings. Future studies should employ clearly defined and reproducible diagnostic criteria, including standardized clinical assessment and, where appropriate, diagnostic medial branch or intra-articular blocks, to more accurately identify patients with facet-mediated pain and determine the efficacy of facet-directed PRP.

Several additional limitations affect the interpretation and clinical application of the current evidence. First, several studies used injections at multiple anatomical sites, which limits attribution of clinical improvement to the facet joint itself. Kirchner et al. [8] administered PRP to multiple spinal structures, including the intradiscal space, facet joints, and transforaminal epidural space. Similarly, Machado et al. [11] evaluated a personalized multi-target approach in which PRP was administered to combinations of the facet joints, intervertebral discs, epidural or foraminal regions, and paravertebral musculature. Although both studies reported improvements in pain and functional outcomes, the simultaneous treatment of multiple potential pain generators prevents attribution of these improvements exclusively to facet joint treatment. Second, follow-up was generally limited, with most studies evaluating outcomes through approximately 6 months and the longest follow-up extending to 12 months. Longer follow-up is needed to determine whether the apparent durability of PRP is sustained.

The available safety data are generally reassuring, with no major safety signal identified in the studies that reported adverse events. Kirchner et al. [8] reported headache, soreness, and local bruising without major adverse events; Machado et al. [11] reported no adverse effects; and Baltzer et al. [7] reported no notable complications. Nevertheless, adverse-event definitions and reporting were inconsistent across studies. Future prospective investigations should therefore use standardized adverse-event definitions and systematic surveillance so that the safety profile of PRP can be evaluated more reliably.

This review itself also has important limitations. The literature search was conducted using PubMed as the sole electronic database, which may have resulted in omission of relevant studies indexed elsewhere and introduced database selection bias. The evidence base was also characterized by small sample sizes, with the largest included study enrolling 86 participants. In addition, efficacy was assessed primarily through patient-reported pain and functional questionnaires, with limited objective outcome measures. These limitations, combined with the small number of comparative studies, reduce the certainty with which the findings can be generalized. Future studies should incorporate objective measures where appropriate, standardized diagnostic criteria, clearly defined injection targets, and clinically meaningful responder thresholds.

From a clinical perspective, the current evidence supports considering PRP as an emerging option for carefully selected patients rather than as an established replacement for conventional facet interventions. PRP may be particularly relevant when the clinical goal is a potentially longer-lasting biologic intervention and when patients understand the uncertainty surrounding benefit. The delayed onset of action associated with a biologic treatment may be a practical disadvantage for patients seeking immediate symptom relief. Finally, cost-effectiveness has not been established, and inconsistent insurance coverage may limit accessibility. At present, the evidence does not justify concluding that PRP is superior to corticosteroid injection for all patients with facet-mediated low back pain. The most important priorities for future research are larger multicenter randomized trials with standardized diagnostic criteria, clearly defined intra-articular or periarticular targets, standardized PRP characterization and dosing, appropriate control groups, systematic adverse-event reporting, and follow-up of at least 12 months. Such studies would help determine whether PRP provides a clinically meaningful advantage in durability and identify which patients and PRP formulations are most likely to benefit.

## 5. Conclusions

In conclusion, the current literature evaluating PRP for facet-mediated mechanical low back pain remains limited but suggests a potential role for PRP in providing sustained improvements in pain and function. However, the available evidence is characterized by small sample sizes, heterogeneous study designs, variable diagnostic criteria, differences in PRP preparation and administration, and limited controlled comparative data. The available controlled studies suggest that PRP may provide more sustained benefits than comparator injections in selected patients, but they do not establish definitive superiority in overall efficacy. In addition, the inclusion of multi-target treatment approaches in some studies limits the ability to attribute observed improvements specifically to facet joint treatment. Therefore, PRP should currently be considered an emerging treatment option rather than an established or proven superior therapy for facet-mediated mechanical low back pain. Larger, well-designed randomized controlled trials using standardized diagnostic criteria, clearly defined facet-directed treatment targets, standardized PRP characterization and dosing, and clinically meaningful outcome measures are needed to better establish its efficacy, durability, and comparative effectiveness.

## Figures and Tables

**Table 1:** Keywords used in search string.

Concept	Line	Keywords
Mechanical Back Pain	1	(“low back pain” OR lumbar”) AND (“non discogenic” OR “mechanical pain”)
Biologics injection	2	“Biologic injections” OR “PRP” OR “Platelet Rich Plasma” OR “Platelet Poor Plasma” OR “Stem Cells” OR “Intramuscular injections” OR “Intraarticular Injections”, “cell component” OR “platelet poor plasma” OR periarticular
Type	3	Intramuscular OR Intraarticular
Combined concepts	4	#1 AND #2 AND #3

PRP: Platelet-rich plasma.

**Table 2:** Comparative characteristics and findings of clinical studies evaluating PRP for lumbar facet-mediated low back pain.

Study/Design (*n*)	PRP Formulation and Injection	Comparator	Outcomes/Follow-Up	Key Findings	Major Limitations
Aufiero et al., 2015 [6] Case series (*n* = 5; 3 lumbar)	Autologous PRP; >1.5 million platelets. Facet joints/surrounding ligaments; 3 injections, 4–6 wk apart.	None	VAS; symptom/function survey. 6–12 mo	Lumbar cases showed substantial pain reduction; VAS 6→1 at 9 mo and 6→2 at 12 mo in reported cases.	Very small; mixed cervical/lumbar population; no comparator; anecdotal evidence.
Kirchner et al., 2016 [8] Retrospective observational (*n* = 86)	PRGF-Endoret. One facet, one intradiscal, and one transforaminal epidural injection.	None	VAS. 1, 3, 6 mo	VAS decreased from 8.4 ± 1.0 to 4.0 ± 2.6, 1.7 ± 2.3, and 0.8 ± 1.7 at 1, 3, and 6 mo.	Retrospective; no comparator; multiple injection sites prevent facet-specific attribution.
Wu et al., 2016 [9] Prospective case series (*n* = 19)	Autologous PRP (~4–5× whole-blood platelet concentration), 0.5 mL; fluoroscopic intra-articular facet injection.	None	VAS, RMDQ, ODI, modified MacNab. 1 wk–3 mo	VAS continued to decrease; RMDQ and ODI significantly improved at 3 mo; 79% reported good/excellent outcomes.	Small; no comparator; short follow-up; incomplete PRP characterization.
Wu et al., 2017 [10] Prospective randomized trial (*n* = 46)	Autologous PRP (~4–5×), 0.5 mL; single intra-articular facet injection.	Local anesthetic/corticosteroid	VAS, RMDQ, ODI, modified MacNab. 1 wk–6 mo	Both groups improved initially; PRP demonstrated more sustained improvement through 6 mo, while comparator benefit declined.	Small sample; 6-mo follow-up; limited PRP characterization; single-center study.
Machado et al., 2022 [11] Prospective case series (*n* = 46)	Leukocyte-poor PRP (~4.17 ± 1.9×); personalized multi-target injections to facets, discs, epidural space and/or paravertebral muscles.	None	VAS, RMDQ. 12, 26, 52 wk	At 12 wk, VAS 8.48→5.17 and RMDQ 18.0→10.98 (*p* < 0.001); improvements sustained to 52 wk.	No comparator; multiple targets confound facet-specific effect; heterogeneous treatment approach.
Baltzer et al., 2025 [7] Prospective controlled, nonrandomized (*n* = 78)	Leukocyte-poor PRP; CT-guided periarticular facet injections; 3–5 weekly injections (mean 3.3).	Local anesthetic: bupivacaine (*n* = 19); PRP *n* = 59	VAS, ODI, VR-12 PCS/MCS. 2 wk–12 mo	At 12 mo, PRP VAS 5.93→2.81 and ODI 23.46→13.65; benefits were sustained compared with LA.	Nonrandomized/self-selected; marked group-size imbalance

Abbreviations: LA, local anesthetic; ODI, Oswestry Disability Index; PRGF, plasma rich in growth factors; PRP, platelet-rich plasma; RMDQ, Roland-Morris Disability Questionnaire; VAS, Visual Analog Scale; VR-12, Veterans RAND 12-Item Health Survey. →: Progressed to.

**Table 3:** Clinical studies methods for creating and administering PRP.

Author	Platelet Count	Injectate Volume	Activated	Leukocytes (Rich/Poor)	System	RBCs	Centrifuge Protocol	Image- Guidance	# of Treatments
Aufiero et al. 2015 [6]	>1.5 million	NR	No	Poor (none)	NR	None	Double spin (no specifics)	Ultrasound Fluoroscopy	3
Kirchner et al. 2016 [8]	2-fold peripheral blood platelet count	4 mL–intradiscal 2 mL–peridural 0.5–facet	PRGF	Leukocyte poor	PRGF System IV	NR	580 g × 8 min	Fluoroscopy	1
Wu et al. 2016 [9]	4–5 × greater than whole blood (100–300 × 10^−9^)	0.5 mL	NR	NR	NR	NR	200 g × 10 min 400 g × 10 min	Fluoroscopy	1
Wu et al. 2017 [10]	4–5× greater than whole blood (100–300 × 10^−9^)	0.5 mL	NR	NR	NR	NR	200 g × 10 min 400 g × 10 min	Fluoroscopy	1
Machado et al. 2022 [11]	4.17 +/− 1.9-fold increase from baseline (Per Machado Turn down-Turn up protocol	Varied: Facet–2 mL Foraminal–2 mL Caudal–5 mL Paravertebral muscles–2 mL Intradiscal–1 mL	NR	Leukocyte poor	Serologic Kasvi centrifuge	Negligible	200 g × 15 min 1600 g × 10 min	Fluoroscopy Ultrasound	1
Baltzer et al. 2025 [7]	NR	NR	NR	Leukocyte Poor	PRP/Arthrex ACP	NR	NR	CT	3–4

NR: Not Reported.

## Data Availability

No new datasets were generated or analyzed in this study. The findings were based on previously published literature cited within the article. Data sharing is therefore not applicable to this article.

## Abbreviations

The following abbreviations are used in this manuscript:

PRP	Platelet-Rich Plasma
PDGF	Platelet-Derived Growth Factor
TGF-B	Transforming Growth Factor-B
IGF	Insulin-Like Growth Factor
VEGF	Vascular Endothelial Growth Factor
EGF	Epidermal Growth Factor
VAS	Visual Analog Scale
PRGF-Endoret	Plasma rich in growth factors
ODI	Oswestry disability index
RMDQ	The Roland–Morris Disability Questionnaire
VR-12	Veteran RAND-12
PCS	Physical Composite Score
MCS	Mental Composite Score
Lp-PRP	Leukocyte-poor platelet-rich plasma
Lr-PRP	Leukocyte-rich platelet-rich plasma
